# Treatment of Philadelphia Chromosome-Positive Acute Lymphoblastic Leukemia in Adults

**DOI:** 10.3390/cancers14071805

**Published:** 2022-04-01

**Authors:** Khalil Saleh, Alexis Fernandez, Florence Pasquier

**Affiliations:** 1 Department of Hematology, Gustave Roussy, 94805 Villejuif, France; khalil.saleh@gustaveroussy.fr (K.S.); alexis.fernandez@gustaveroussy.fr (A.F.); 2 INSERM, UMR 1287, Gustave Roussy, Université Paris-Saclay, 94805 Villejuif, France

**Keywords:** acute lymphoblastic leukemia, tyrosine kinase inhibitors, monoclonal antibodies, Philadelphia chromosome

## Abstract

**Simple Summary:**

Outcome of patients with Philadelphia-chromosome positive acute lymphoblastic leukemia (Ph+ ALL) dramatically improved during the past 20 years with the advent of tyrosine kinase inhibitors and monoclonal antibodies. Their great efficacy in young and fit patients led to question our reliance on chemotherapy and allogeneic hematopoietic stem cell transplantation. Moreover, these well-tolerated treatments can be safely administrated even in the elderly that represent the majority of Ph+ ALL patient. This review will focus on the recent changes of paradigm in the management of Ph+ ALL patients and the development of novel therapeutic strategies.

**Abstract:**

Philadelphia-chromosome positive acute lymphoblastic leukemia (Ph+ ALL) is the most common subtype of B-ALL in adults and its incidence increases with age. It is characterized by the presence of BCR-ABL oncoprotein that plays a central role in the leukemogenesis of Ph+ ALL. Ph+ ALL patients traditionally had dismal prognosis and long-term survivors were only observed among patients who underwent allogeneic hematopoietic stem cell transplantation (allo-HSCT) in first complete remission (CR1). However, feasibility of allo-HSCT is limited in this elderly population. Fortunately, development of increasingly powerful tyrosine kinase inhibitors (TKIs) from the beginning of the 2000′s dramatically improved the prognosis of Ph+ ALL patients with complete response rates above 90%, deep molecular responses and prolonged survival, altogether with good tolerance. TKIs became the keystone of Ph+ ALL management and their great efficacy led to develop reduced-intensity chemotherapy backbones. Subsequent introduction of blinatumomab allowed going further with development of chemo free strategies. This review will focus on these amazing recent advances as well as novel therapeutic strategies in adult Ph+ ALL.

## 1. Introduction

Acute lymphoblastic leukemia (ALL) is the most common childhood cancer but it can affect patients of any age. The genetic landscape of ALL is heterogeneous and varies with age. Philadelphia-chromosome is the most common cytogenetic abnormality among adult ALL patients, accounting for 25% of cases with incidence increasing to more than 40% in the elderly [1,2]. Philadelphia-chromosome positive (Ph+) ALL is characterized by the presence of reciprocal translocation t(9;22)(q34;q11), leading to BCR-ABL fusion gene encoding BCR-ABL oncoprotein, that has constitutive tyrosine kinase activity and plays a central role in ALL development [3,4]. BCR-ABL is a crucial biomarker for the diagnosis of Ph+ ALL and the monitoring of minimal residual disease (MRD). BCR-ABL is also a therapeutic target for tyrosine kinase inhibitors (TKIs) [5].

Before the TKIs era, the prognosis of Ph+ ALL patients was poor with almost no long-term survivors apart from patients who underwent allogeneic hematopoietic stem cell transplantation (Allo-HSCT) [6,7,8,9]. Fortunately, the outcome of Ph+ ALL patients has dramatically improved over the past 20 years with the introduction of TKIs. TKIs are small oral molecules that competitively block the binding of ATP to the ATP-binding domain of BCR-ABL and inhibit downstream signal transduction pathway. Imatinib was the first available TKI, followed by second generation (2G—dasatinib, nilotinib, bosutinib) and third generation (3G—ponatinib) TKIs who have stronger and faster activity against BCR-ABL. 2G/3G-TKIs overcome several ABL mutations, which are involved in treatment resistance and relapse. More recently, development of monoclonal antibodies like blinatumomab led to the development of chemotherapy-free strategies with very promising results and excellent tolerance. 

This review will focus on the current standards and recent advances in the management of adult patients with Ph+ ALL including impact of frontline first generation (1G) versus 2G/3G-TKI administration on the intensity of remission and post-remission treatments, novel chemotherapy-free strategies based on blinatumomab administration as well as salvage treatment with the development of new therapeutic approaches.

## 2. Frontline Treatment of Adult Ph ALL Patients

In the pre-TKIs era, complete response (CR) was less frequent than for Ph negative (Ph-) ALL patients with CR rates lower than 70% and long-term OS less than 20% with different outcomes according the post remission treatment. The best results were obtained for allo-HSCT recipients with long-term survival of 40% to 50%, illustrating the graft versus leukemia (GvL) effect in Ph+ ALL [6,7,10,11,12,13,14]. Intensive chemotherapy followed by allo-HSCT became the standard of care in Ph+ ALL patients but donor availability and treatment related mortality (TRM) limit its feasibility in adult population, underlining the need for new approaches in the management of Ph+ ALL [7,8,9]. Development of targeted therapy against BCR-ABL, imatinib and subsequent 2G/3G-TKIs, and more recently of blinatumomab improved response to treatment and survival of adult patients with Ph+ ALL. Table 1 resumes prospective trials conducted in adult Ph+ ALL patients in the TKIs era, according to the intensity of the chemotherapy backbones.

### 2.1. Intensive Chemotherapy plus TKIs

#### 2.1.1. Imatinib plus Intensive Chemotherapy

Imatinib showed in the pivotal IRIS study remarkable efficacy with high rate of durable response and long-term survival in patients with chronic myeloid leukemia in chronic phase (CML-CP) [15,16]. Imatinib as single agent induced high remission rate in Ph+ ALL patients but this benefit did not translate into longer survival [17]. Therefore, several phase 2 and 3 studies evaluated frontline imatinib with intensive chemotherapy in newly diagnosed Ph+ ALL patients [7,18,19,20,21,22,23]. Concurrent administration of imatinib and chemotherapy had greater anti-leukemic effect than alternating administration schedule [24]. CR rate increased significantly compared to pre-TKI era (above 90%). Overall complete molecular remission (CMR) rates were less than 50%. The 5-year overall survival (OS) rates ranged from 43% to 50% and 5-year event free survival (EFS) or disease-free survival (DFS) rates from 32% to 52% (Table 1). Retrospective analyses found significant improvement of response and long-term outcome with imatinib administration in comparison with the pre-TKIs era [6,7,14,19,20,23,25,26,27,28]. Prospective randomized trials with or without TKIs cannot be performed due to the obvious effect of TKIs. These excellent results are in part due to high CR rate obtained with imatinib allowing more patients to undergo allo-HSCT. The percentage of patients undergoing allo-HSCT increased to more than 50% for imatinib recipients in first complete remission (CR1) and absolute number of allo-HCST for Ph+ ALL patients increased during the TKI era (+166% between 2001–2003 and 2013–2015) [6,7,14,18,19,23,29]. Moreover, imatinib administration in itself also improves the long-term outcome of Ph+ ALL patients independently of the post remission treatment [6,7,14,19,20,23,25,26,30]. In a retrospective study from the European Society for Blood and Marrow Transplantation (EBMT), pre-transplant TKI administration was associated with significant better OS survival (HR = 0.68; *p* = 0.04) and lower cumulative incidence of relapse (CIR) (HR = 0.5; *p* = 0.01) in multivariate analysis [26]. Imatinib administration also improves long-term outcome of patients receiving chemotherapy only while no long-term survivors were observed in the pre-TKIs era [22,23,31].

#### 2.1.2. 2G/3G-TKIS plus Intensive Chemotherapy

Given the good results with imatinib, 2G/3G-TKIs were developed. They have superior potency as inhibitors of BCR-ABL than imatinib and activity against BCR-ABL mutations [32,33,34,35]. They induce rapid and deep responses in CML shown in various phase 2 or 3 trials and are approved in this indication as first line treatment or beyond [36,37,38,39,40]. In three phase 2 studies in Ph+ ALL, CR rates with dasatinib or nilotinib in combination with intensive chemotherapy were 96%, 91% and 100%, respectively [41,42,43]. Overall CMR rates were 65% with dasatinib and 86% with nilotinib. Long-term OS and DFS were above 50% (Table 1) [41,42,43]. There is actually no data on bosutinib in first-line setting. Ponatinib is the only 3G-TKI available and the most powerful one with fast and deep molecular responses [35]. Ponatinib overcomes the T315I mutation that is present in up to 75% of relapsing patients after imatinib or 2G-TKIs [44]. Ponatinib was evaluated in association with the HCVAD regimen in adult Ph+ ALL patients in one phase 2 trial. After two ponatinib-related deaths from myocardial infarction, the protocol was amended to use lower dosage of ponatinib and no further death occurred. CR rate was 100%, CMR was obtained in 83% of cases at any time with median time to CMR of 3 months, translating into 5-year OS of 73% and 5-year EFS of 68% (Table 1) [45,46]. Overall, frontline 2G/3G-TKIs administration with intensive chemotherapy improved the deepness of the response and the long-term outcome of adult patients with Ph+ ALL, translating into lower allo-HCST rates in Ravandi et al. (17%) and Jabbour et al. (20%) [20,29,41,45,47]. Therefore, the great efficacy of 2G/3G-TKIs could challenge allo-HSCT as gold standard post-remission treatment. Comparative prospective studies are required to confirm the great results of these phase 2 clinical trials. 

### 2.2. De-Intensification of Chemotherapy

The global good tolerance of concomitant administration of TKIs plus intensive chemotherapy should however not hide potential serious adverse events (AEs). Early induction deaths led to amend several protocols to reduce TKI dosage or chemotherapy intensity [26,45]. In addition, Ph+ ALL patients are older than Ph- ALL patients and often unfit for intensive chemotherapy due to comorbidities. Given the great efficacy of frontline TKIs administration and the subsequent development of blinatumomab, several cooperative groups evaluated de-intensification of chemotherapy backbones, even in younger and fit patients (Table 1). 

#### 2.2.1. Low Intensity Chemotherapy plus TKIs

The European Working Group for Adult Acute Lymphoblastic Leukemia (EWALL) evaluated the combination of 2G-TKIs with low intensity chemotherapy (weekly vincristine IV and oral dexamethasone for 2 days for 4 weeks with central nervous system (CNS) prophylaxis, V-D backbone) in Ph+ ALL patients aged 55 years and older. The EWALL-Ph-01 trial enrolled 71 patients and evaluated the combination of dasatinib with V-D induction [48] and the EWALL-Ph-02 trial evaluated in 72 patients the combination of V-D backbone with nilotinib [49]. The CR rates were 96% in the EWALL-Ph-01 and 94.4% in the EWALL-Ph-02 but only 25% and 58% of patients achieved CMR after two chemotherapy courses, respectively. In the EWALL-Ph-01 trial, the 5-year OS rate was 36% and the 5-year EFS rate was 27%. In the EWALL-Ph-02 trial, the 4-year OS rate was 47% and the 4-year EFS rate was 42%. Relapses were in both trials the main cause of treatment failure, reflecting an insufficient control of the disease (Table 1). The combination of TKIs with low intensity chemotherapy was also evaluated in younger patients (Table 1). Thirty-five Ph+ ALL patients enrolled in the GMALL08/2013 phase 3 trial had a CR rate of 95% and a 3-year OS rate of 74% [50]. The Group for Research on Adult Acute Lymphoblastic Leukemia (GRAALL) conducted in France and in Switzerland a randomized phase 3 trial (GRAAPH 2005) in patients aged 18 to 59 years with newly diagnosed Ph+ ALL to compare intensive chemotherapy versus low intensity chemotherapy (V-D backbone) as induction treatment. All patients received imatinib. CR rate was significantly higher in the low intensity arm (98.5% vs. 91%, *p*= 0.006), due to fewer induction deaths. Molecular responses were similar between both arms, demonstrating the non-inferiority of the low intensity arm [18]. CMR rates were 9.5% after induction and 26.8% after the second course of chemotherapy, and 5-year OS and DFS were 43% and 42% respectively [18]. The subsequent GRAAPH 2014 trial combined Nilotinib to the V-D induction backbone. CR rate was similar to that of GRAAPH 2005 (98%) but CMR rate raised above 70% after 4 cycles of chemotherapy and 3-year OS and DFS rates were 86% and 79.6% respectively, in the control arm [51,52]. However, omission of high-dose cytarabine during consolidation treatment in the experimental arm was associated with increased risk of relapse due to emergence of T315I mutations, despite non-inferior levels of late molecular responses, translating into lower DFS and OS [52]. Several ongoing trials are evaluating reduced-intensity chemotherapy in combination with TKIs (Table 2).

Moreover, to reduce the initial treatment toxicity, several phase 2 trials evaluated chemotherapy-free induction regimens based on steroids and TKIs administration as an alternative to systemic chemotherapy for patients younger than 60 years and fit for intensive treatment [26,53,54,55,56,57]. Patients in CR1 received subsequently chemotherapy with TKIs and allo-HSCT if eligible. CR rates were >95% for most of these trials, with no or few deaths in induction, and irrespective of the administered TKI (imatinib or dasatinib). Long-term survival seems to be improved with dasatinib compared to imatinib administration, probably due to deeper molecular response [54,56]. However, relapses remain a matter of concern, especially for patients who did not undergo allo-HSCT. T351I positive relapses accounted for 71% of relapses in the GIMEMA 1205 trial [26,53]. 

#### 2.2.2. Chemotherapy-Free Strategies

##### Steroids plus TKIs Regimens

To reduce overall treatment toxicity in elderly patients, the GIMEMA cooperative group developed chemotherapy-free strategies based on the combination of steroids and TKIs for elderly and/or unfit patients. Several phase 2 trials were conducted either with imatinib 800 mg/d in the GIMEMA 0201-B trial, alternating courses of nilotinib 400 mg/d and imatinib 600 mg/d in the GIMEMA 1408 trial, or ponatinib 45 mg/d in the GIMEMA 1811 trial [58,59,60]. As well as for younger patients, steroids plus TKIs led to CR rates of 95% or above with no induction deaths with imatinib and nilotinib [58,59]. In the GIMEMA 1811 trial, 2 patients died during induction but both deaths were considered unrelated to the study treatment [60]. As observed in younger patients, these phase 2 clinical trials confirmed the feasibility and the safety of steroids plus TKI induction in older or unfit patients. However, these high remission rates did not translate into prolonged survival, because of low CMR rates [58,59] (Table 1). Long-term results of the GIMEMA 1811 are pending.

##### Binatumomab Based Regimen

Steroids plus TKIs chemotherapy-free regimen induces high CR rates with few early deaths but duration of response remains short and relapses are still a major issue, especially in case of T315I mutation, underlining the need for a better control of the disease.

Blinatumomab is a bispecific T-cell engager that binds to both CD19 on B ALL cells and CD3 on T cells. This interaction leads to the activation and proliferation of T cells that can exert cytotoxic effect against CD19+ leukemic cells. In the setting of Ph- B-ALL, Blinatumomab has demonstrated its superiority over chemotherapy in relapsed and/or refractory (R/R) patients as well as in MRD positive patients with low side effects [61,62,63,64]. As its mechanism of action is independent from BCR-ABL fusion protein, directing cytotoxic T cells to leukemic blasts, blinatumomab can overcome BCR-ABL mutations including T315I mutation [65]. Given its efficacy and its excellent tolerance in R/R ALL patients, the GIMEMA incorporated blinatumomab as consolidation treatment in the LAL 2116 (D-ALBA) trial [66,67]. Adult patients with no upper age limit and with newly diagnosed Ph+ ALL were eligible. After a 7 days pre phase with steroids, patients received steroids for 24 more days and dasatinib for 85 days followed by at least 2 cycles of blinatumomab with dasatinib. Twelve prophylactic CNS lumbar punctures were performed. Twenty-nine patients (50%) underwent allo-HSCT after at least one cycle of blinatumomab. At the end of induction, 98% of patients were in CR and 29% had a molecular response. The percentage of patients with molecular response further increased with blinatumomab administration: 64% of patients had molecular response (CMR: 35%) after 1 cycle of blinatumomab and 72% (CMR: 55%) after 5 cycles. At the last follow-up, the 2-years OS rate was 87.8% and 2-year DFS rate was 79.8%. Patients achieving molecular response and with no IKZF1 deletion had significant better DFS rate. The most common adverse events of grade 3 or higher were unexpected CMV reactivations in five patients. Nine patients relapsed with four relapses isolated in the CNS. In the first report of the study, among the six patients who relapsed, five carried a T315I mutation. 

The SWOG Cancer Research Network reported at the 2021 American Society of Hematology (ASH) congress the outcome of 25 patients enrolled in the SWOG 1318 trial [68]. They received similar regimen than in the D-ALBA study. After induction, 92% of patients achieved CR and 31% of patients achieved CMR. The estimated 3-year OS and DFS rates were 85% and 80%, respectively. Tolerance was good with no early death, two non-hematologic grade 4 toxicities during induction, and no grade 4 or higher treatment-related non-hematologic toxicities subsequently. 

The MD Anderson Cancer Center (MDACC) conducted a single-arm phase 2 study of blinatumomab plus ponatinib in 19 newly diagnosed Ph+ ALL patients with median age of 62 years (range, 34–83) [69]. Blinatumomab was infused from induction (that is to say earlier than for the D-ALBA and SWOG 118 trials) and up to 5 cycles in combination with ponatinib 30 mg daily initially and 15 mg daily once CMR was achieved, followed by 5 years of ponatinib maintenance therapy. CR/CR with incomplete hematologic recovery (CRi) was achieved in 100% of patients and CMR in 87% of patients. No patient underwent allo-HSCT. The 1-year OS and EFS rates were both 100%. The tolerance was good with only one patient discontinuing treatment due to side effects (recurrent grade 2 tremor with blinatumomab).

Overall, the chemotherapy-free strategies combining TKIs and blinatumomab are efficient and well tolerated even in older/unfit patients. Side effects of blinatumomab are well known (cytopenias, hepatotoxicity, cytokine release syndrome, neurologic events) and manageable, with few treatment interruptions. Long-term results of these phase 2 studies are pending. Several clinical trials are ongoing to further evaluate chemotherapy-free regimen in Ph+ ALL patients (Table 2).

## 3. Allogeneic Hematopoietic Stem Cell

Since the pre-TKIs era, allo-HSCT is considered as the only curative treatment and the standard of care for adult patients with Ph+ ALL in CR1. As previously mentioned, addition of TKIs to frontline treatment dramatically improved response rate, allowing more patients to obtain MRD negativity before allo-HSCT, which is correlated to reduced relapse risk [27,70,71,72]. Here we discuss how TKI administration led to question the role of allo-HSCT in the management of adult patients with Ph+ ALL.

### 3.1. Allo-HSCT in the TKIs Era

There are actually no randomized trials evaluating the different post-remission strategies in Ph+ ALL patients. Yet, several prospective trials reported the outcome of Ph+ ALL patients according their post remission treatment: allo-HSCT, chemotherapy alone or, to a lesser extent, autologous HSCT (auto-HSCT). As in the pre-TKIs era, most of the data show survival benefit for imatinib recipients undergoing allo-HSCT (Table 3) [7,18,20,26,73]. However, some studies did not find any significant survival advantage for allo-HSCT in the setting of imatinib administration as imatinib improved long-term survival in patients receiving only chemotherapy [21,22,23,31]. In a randomized pediatric/adolescents and young adults (AYA) trial, patients treated with imatinib and chemotherapy had the same 5-year DFS and EFS rates than patients undergoing allo-HSCT [74]. The survival benefit of allo-HSCT in the setting of 2G and 3G TKIs is less clear and a detrimental effect of allo-HSCT related to TRM was even observed (Table 3) [21,41,43,45]. In the phase 2 study combining HCVAD plus dasatinib, only patients under 40 years old still benefit from allo-HSCT in CR1 [41]. After frontline treatment with HCVAD and ponatinib, Short et al. reported a 3-year OS rate of 66% for allo-HSCT recipients (*n* = 18) versus 90% for patients who did not undergo allo-HSCT (*n* = 57; *p* = 0.07) [46]. The allo-HSCT rate was particularly low (17% and 20%) in these studies [41,45]. These data are supported by two recent meta-analyses that compared efficacy of allo-HSCT to chemotherapy or auto-HSCT [31,75]. Analysis combining data from pre-TKIs and TKIs eras found significant survival benefit of allo-HSCT due to lower CIR, but with higher TRM rates. When analysis was restrained to TKIs recipients (mostly imatinib), odds ratio of OS and DFS between allo-HSCT and chemotherapy groups decreased. Moreover, when analysis was restrained to 2G and 3G-TKI, there was no more survival advantage for allo-HSCT compared to chemotherapy as post remission treatment [31]. Altogether, these results demonstrate survival advantage for allo-HSCT in the imatinib setting even if this benefit seems less significant than in the pre-TKIs era. This advantage seems to vanish with 2G/3G-TKIs due to their greater efficacy compared to imatinib and the TRM rate of allo-HSCT [22,30,41]. However, most of these data come from phase 2 studies with small number of patients. Prospective comparative trials to clarify the role of allo-HSCT in the context of 2G/3G-TKIs and blinatumomab are required. Allo-HSCT remains the preferred curative approach for eligible adult patients with Ph+ ALL in CR1 in the current clinical practice.

### 3.2. Myelo-Ablative Conditioning (MAC) versus Reduced-Intensity Conditioning (RIC)

Allo-HSCT prevents relapses through its GvL effect but also the intensity of the conditioning in case of MAC allo-HSCT. Total body irradiation (TBI) based conditioning is the gold standard in ALL, as it was associated with reduced relapse risk in numerous retrospective studies [76]. Moreover, in a recently published pediatric randomized phase 3 study, TBI conditioning was associated with improved OS and lower risk of relapse compared with chemotherapy-based conditioning [77]. TRM rate limits its feasibility particularly in old or unfit patients, thus majority of Ph+ ALL adult patients are not eligible to MAC allo-HSCT. RIC was developed to allow patients that are unfit for MAC to receive allo-HSCT and potentially benefit from the GvL effect with reduced TRM. Feasibility and efficacy of RIC before allo-HSCT in Ph- ALL patients has been demonstrated in several trials [78,79,80]. Pre- and post-transplant TKI administration and the lower TRM of RIC offset the increased relapse risk observed with RIC compared to MAC [27,47,81] leading to similar OS and DFS or leukemia-free survival (LFS) between RIC and MAC recipients [18,82,83,84]. In Bachanova et al., RIC recipients with TKI administration and negative MRD before allo-HSCT had even a significant better OS than patients with similar pre-allo HSCT MRD undergoing MAC allo-HSCT (55% vs. 33%, *p* = 0042%) [27]. MAC remains the standard of care for younger patients and RIC is a potent alternative for older or unfit patients, particularly in situation of negative MRD before allo-HSCT.

### 3.3. Post Allo-HSCT Maintenance

Relapse after allo-HSCT is a common reason for treatment failure and post-transplantation administration of TKIs is a strategy to prevent Ph+ ALL recurrence. Several studies (including one randomized trial) had evaluated administration of TKIs (mainly imatinib) after allo-HSCT [24,27,47,81,85,86,87,88,89,90,91,92,93,94,95,96,97,98,99,100,101,102,103,104,105,106]. Most of them found a positive impact of post-transplant TKIs administration on OS and CIR rates [27,47,81,87,88,89,90,100,102,104,107] and no association between TKIs’ administration and incidence of graft versus host disease [108]. These studies have limitations as small number of patients, heterogeneous cohort (patients in CR1 and beyond), limited follow-up and variable dosing, starting date and duration of TKI administration.

The EBMT and the 2021 National Comprehensive Cancer Network (NCCN) guidelines recommend starting TKIs administration for all Ph+ ALL patients as soon as possible after full hematologic recovery from allo-HSCT. Recommended dosage is lower than in the pre-transplantation period in order to reduce toxicities (400 mg/d for Imatinib, 50–100 mg/d for Dasatinib, 200mg bid for Nilotinib and 15 mg/d for Ponatinib) [106,109]. Imatinib administration should be favored except in case of imatinib resistance in the pre-alloSCT period, pre-existing ABL kinase domain mutation or in the context of CNS involvement as imatinib poorly penetrates CNS, contrary to dasatinib or ponatinib [108,110,111,112]. Regular MRD monitoring is required [104]. In case of detectable MRD, BCR-ABL mutation analysis must be performed [113]. The minimal duration of post-transplantation TKIs administration is 1 year with continuous undetectable MRD for patients in CR1 and indefinitely for patients in CR2 and beyond, even if the optimal duration is unknown [106,113]. 

Whether preemptive or prophylactic strategies should be favored remains undefined [104,107,114,115]. A preemptive strategy implies a strict and frequent MRD monitoring with rapid results to allow a quick intervention if needed [106,107]. 

To note, a recent phase 2 study demonstrated the feasibility and safety of post allo-HSCT blinatumomab based maintenance in 21 patients including 2 Ph+ ALL patients [116]. Several prospective trials are ongoing to clarify the implementation of post-allo HSCT maintenance (Table 2). 

## 4. Auto-HSCT

Auto-HSCT plus maintenance is an alternative to allo-HSCT as post-remission therapy with less TRM but increased relapse risk. In the pre-TKIs era, auto-HSCT did not improve patients’ outcome compared to chemotherapy alone [117,118]. Rapid and deep response before auto-HSCT decreases CIR and improves long-term outcome, reflecting the importance of “cleaning” bone marrow from leukemic cells before stem cell mobilization [117,119,120]. Thus, addition of TKI to the frontline treatment allowed performing auto-HSCT in better conditions than in the pre-TKI era. This is illustrated by an EBMT study among 177 Ph+ ALL patients receiving auto-HSCT in CR1 where long-term outcome was significantly improved in the TKI era (3-year OS was 16% between 1996 and 2001, 48% between 2002 and 2006 and 57% between 2007 and 2010, *p* < 0.0001; 3-year LFS was 11%, 39%, and 52%, respectively). The 3-year LFS of patients achieving deep molecular response before auto-HSCT increased to up to 65% [121]. In small series of highly selected patients, long term outcome was similar among patients undergoing allo-HSCT and auto-HSCT [18,19,20,120,122]. Relapses after auto-HSCT remain a limiting factor and maintenance with TKI in the post-transplant period is crucial. Use of auto-HSCT decreases over time, but between 2013 and 2015, Ph+ ALL patients still represented 47% of auto-HSCT recipients [29]. Thus, auto-HSCT is an option in selected population with deep molecular response before transplantation and contraindication to allo-HSCT. However, given the great efficacy of 2G/3G-TKIs and blinatumomab, it is likely that auto-HSCT will no longer be considered as state of the art therapy option.

## 5. Treatment of Relapsed/Refractory Ph+ ALL Patients

Despite high remission rate and prolonged survival compared to the pre-TKIs era, relapses remain a major cause for treatment failure. Recurrent measurable MRD is associated with high relapse rate and poor survival and regular MRD monitoring is necessary after completion of frontline treatment [106]. Relapses are often due to BCR-ABL kinase domain resistance mutations, which are found in 70% to 80% of imatinib resistant patients and in 80% of 2G-TKIs resistant patients [123,124,125]. The NCCN recommend performing BCR-ABL mutation screening in R/R Ph+ ALL patients [109]. The most frequent mutation in Ph+ ALL patients is the T315I mutation, which is resistant to all TKIs except ponatinib [126], followed by the P-loop mutations E255K and Y253H. In Soverini et al., these 3 mutations were detected in 75% of imatinib resistant patients. The T315I mutation was detected in 80% of dasatinib resistant patients [125]. Allo-HSCT remains the only curative treatment in the relapse setting. Low CR rate as well as poor long-term outcome can be expected with conventional chemotherapy as salvage therapy [127,128]. Here we focus on the development of novel anti leukemic agents in the setting of relapsed or refractory (R/R) Ph+ ALL patients.

### 5.1. Ponatinib as Single Agent

Ponatinib (45 mg daily) was evaluated as single agent in the context of R/R Ph+ ALL. Among the 447 patients included in the PACE phase 2 clinical trial, 32 patients had R/R Ph+ ALL. Major hematologic response was obtained in 41% of cases with a median time to response of 2.9 weeks. The estimated rate of sustained response of at least 12 months was 8%. Complete cytogenetic response was observed in 38% of cases. The estimated 1-year PFS and OS rates were 7% and 40% respectively [40]. One Japanese phase 1/2 study and the multicenter observational retrospective OPAL trial confirmed the efficacy and tolerability of ponatinib as single agent for R/R Ph+ ALL patients [129,130]. Ponatinib was further evaluated in association with new anti-leukemic agents.

### 5.2. Blinatumomab as Single Agent

As for Ph- B-ALL patients, blinatumomab was evaluated as single agent for the treatment of R/R Ph+ ALL in the ALCANTARA phase 2 multicenter study [65,131,132]. The study enrolled 45 patients with R/R Ph+ ALL with median age of 55 years (range, 23–78). They all had received prior TKIs and 51% of them had received prior ponatinib. Thirty-one percent of patients achieved CR after one cycle of blinatumomab infusion. Among them, 86% achieved CMR regardless of prior TKI therapy or BCR-ABL kinase domain mutations. Among 10 patients with T315I, 4 (40%) achieved CR and CMR. Median relapse-free survival (RFS) was 6.8 months after a median follow-up of 16.1 months and median OS was 9.0 months with a median follow-up of 25.1 months. Twenty-five percent of patients underwent allo-HSCT while on remission after blinatumomab. Tolerance was good with only one patient requiring treatment interruption for aphasia. A propensity score analysis comparing data from the ALCANTARA study to those of an external population of R/R Ph+ patients who had received standard of care as salvage therapy suggested a trend for better CHR rate and significant longer survival for the blinatumomab cohort [133]. Blinatumomab was approved in July 2017 for the treatment of R/R B-ALL in adult and children.

### 5.3. Inotuzumab Ozogamicin (INO) as Single Agent

INO is a humanized anti-CD22 monoclonal antibody conjugated to a cytotoxic agent that induces apoptosis after binding to CD22 and internalization. In the INO-VATE ALL phase 3 trial, adults with R/R Ph+ and Ph- B-ALL were randomized to receive INO or investigator’s choice of chemotherapy. Patients in the INO arm had significant higher CR rate, higher rate of molecular response, and longer PFS and OS than patients in the control arm. However, when analysis was restricted to Ph+ ALL patients, the study failed to prove superiority of INO over chemotherapy [134]. In a post-hoc analysis of the 22 Ph+ ALL patients enrolled in the INO arm, 73% achieved CR/CRi, 41% underwent allo-HSCT and median PFS and OS were 3.9 months and 8.7 months respectively. Again, despite higher rate of MRD negativity among Ph+ ALL patients in CR/CRi in the INO group compared to the chemotherapy group (81% vs. 33%, *p* = 0.009), it did not translate into longer OS or PFS, confirming the results of the initial report [135]. Based on these data, INO was approved by the Food and Drug Administration (FDA) in 2017 for the treatment of adults with R/R B-ALL, including patients with Ph+ ALL. INO is associated with increased risk of veno-occlusive disease (VOD), a potentially life-threatening complication due to sinusoidal cell damage leading to post sinusoidal portal hypertension. An expert panel proposed recommendations for the management of VOD during INO [136,137].

### 5.4. Ponatinib Plus Blinatumomab

The phase 2 study of ponatinib and blinatumomab previously mentioned in this issue in the context of newly diagnosed Ph+ patients also enrolled 14 patients with R/R Ph+ ALL with median age of 38 years (range, 24–61). CR/CRi rate was 91% and CMR rate was 82% after one cycle of treatment. The estimated 2-year EFS and OS rates were 53% and 39%, respectively. The tolerance was correct with no early death and most side effects of grade 1–2 [138]. In a retrospective study on 26 patients with R/R Ph+ ALL treated with the combination of blinatumomab and ponatinib, CR and CMR rates were 96.2% and 88.5%, respectively. Thirty-two percent of the patients of patients proceeded to allo-HSCT. The 2-year OS and EFS rates were 41.4% and 31.8% [139]. The combination of blinatumomab and a TKI (either 2G or 3G-TKI) was also reported in 3 smaller studies with reasonable clinical outcomes [140,141,142]. 

### 5.5. Ponatinib Plus Venetoclax

Several pre-clinical studies suggested that dual targeting of pro-survival BCL2 signaling and BCR-ABL with the combination of venetoclax and a TKI may have an anti-leukemic effect and prevent resistance in the Ph+ ALL setting [143,144,145]. Combination of ponatinib, venetoclax and dexamethasone (VPD regimen) was evaluated in a phase 1/2 clinical trial that enrolled 9 patients with R/R Ph+ ALL [146]. Three patients received venetoclax at the dosage of 400 mg daily and 6 patients at the dosage of 800 mg daily. No dose-limiting toxicity was observed. The most frequent adverse events leading to dose reduction or treatment interruption were myelosuppression, elevation of transaminases and deep vein thrombosis/pulmonary embolism in 1 patient. Four patients (44%) achieved CR and CMR; they all received venetoclax at the 800 mg daily dose. No patient treated with 400 mg daily dose achieved response. Median OS was not reached with a median follow-up of 13.2 months.

Outcome of 19 patients with T315I/compound-mutated R/R Ph+ ALL treated with the VPD regimen (venetoclax 400 mg daily) was reported at the 2021 ASH congress [147]. After 1 cycle, 17 patients (89.5%) achieved CR/CRi and 42% achieved CMR. Subsequent relapses occurred in eight patients (41%). Among relapsed patients, one had proceeded to allo-HSCT and seven continued VPD regimen. Grade 3–4 adverse events were mainly myelosupression and infections. To note, 7/19 patients were treated as outpatients. These preliminary results support preclinical data suggesting a synergistic effect of venetoclax and TKIs on Ph+ ALL.

### 5.6. Inotuzumab Ozogamicin + Bosutinib

A phase 1/2 trial combining INO and bosutinib was conducted in patients with R/R Ph+ ALL (*n* = 18) and R/R CML in lymphoid blast phase (*n* = 2) with median age of 62 years (range, 19–74). Patients with T315I mutation were excluded. The CR rate was 61% and the CMR rate was 56%. At a median follow-up of 44 months, median EFS and OS were 7.7 and 13.5 months, respectively. The tolerance was good with no early death and no VOD. The maximum tolerated dose of bosutinib was 400 mg daily [148]. The phase 2 portion of the trial is ongoing.

### 5.7. Asciminib Plus TKIs

Asciminib is first-in-class selective allosteric ABL myristoyl pocket (STAMP) inhibitor restoring the negative regulator functions of ABL. Contrary to conventional TKIs, asciminib do not bind BCR-ABL ATP-binding site and is active against several resistance mutations, such as T315I. Asciminib demonstrated significant superior efficacy compared with bosutinib alone with a favorable safety profile in the ASCEMBL phase 3 study in patients with CML-CP previously treated with ≥2 TKIs [149]. In a phase 1 study, asciminib showed efficacy in the setting of CML-CP patients with T315I mutation with MMR rates at week 24 and week 96 of 40.8% and 46.9%, respectively [150]. In October 2021, asciminib was granted accelerated approval for the treatment of adults with CML-CP previously treated with ≥2 TKIs, and full approval for the treatment of adults with CML-CP with T315I mutation [151]. In a phase 1 study that enrolled 12 untreated adult patients with Ph+ ALL, the combination of asciminib, dasatinib and prednisone was feasible with asymptomatic pancreatic enzyme elevation as primary toxicity. All patients (10/10) obtained CR after one month of treatment. After 3 months of treatment, all evaluable patients (7/7) remained in CR and 71% of them had a BCR-ABL 3-log reduction [152]. Preclinical data support the combination of asciminib and ponatinib and one case report has illustrated its feasibility [153,154].

### 5.8. Chimeric Antigen Receptor T Cells

Chimeric antigen receptor (CAR) T-cells are genetically engineered to express cell surface CAR that recognizes antigen on leukemic cells and induces cell death. CARs are artificial fusion proteins combining an extracellular antigen-binding domain linked to intracellular T-cell signaling domains and co-stimulatory molecules. CAR T-cells recognize specific antigen and are activated independently of the major histocompatibility complex [155,156,157]. As CD19 is expressed on nearly all B-ALL cells, it was considered as an ideal target leading to the development of anti-CD19 CAR T-cells.

Tisagenlecleucel, an autologous anti-CD19 CAR T-cell therapy, induced high CR rates in a pilot phase 1/2 trial with sustained responses in 30 pediatric and AYA R/R B-ALL patients [158]. These promising results were confirmed by the ELIANA phase 2 trial, which enrolled 75 children and AYA patients with R/R B-ALL. The CR rate was 81% and 1-year OS and DFS were 76% and 50% [159]. FDA and EMA approved Tisagenlecleucel for the treatment of pediatric and young adult patients with B-ALL that is refractory or in second or later relapse. Several patients with Ph+ ALL were enrolled in these trials but no subgroup analysis is available.

Brexucabtagene autoleucel (KTE-X19) is another autologous CD19-targeting CAR T-cells evaluated in the ZUMA-3 phase 2 clinical trial that enrolled 71 adults R/R B-ALL patients including 27% with Ph+ ALL [160]. Fifty-five patients actually received the treatment. Median age was 40 years (range, 28-52). The CR/CRi rate was 71% and among responders, 97% obtained CMR. At 6 months, RFS was 58% and the 1-year OS rate was 71%. Among the 15 Ph+ ALL patients, CR rate was 80%, 6-month RFS rate was 60% and 1-year OS rate was 93%. Thirty-eight percent of the patients proceeded subsequently to allo-HSCT. FDA approved brexucabtagene autoleucel in October 2021 for adult patients with R/R B-cell precursor ALL.

To improve efficacy of CAR T-cells, dual therapy that targets CD19 and CD22 has been established with promising results (Table 2) [161,162]. 

CAR T-cells are associated with severe side effects that can be life-threatening and consensus guidelines for the management of cytokine release syndrome and neurotoxicity were published in 2018 [163]. Management of CAR T-cells’ toxicity massively improved over the last years. The advent of CAR T-cells therapy offers a highly promising treatment option and leads to unprecedented and impressive results in patients with B-cell malignancies. Nevertheless, its role in the management of Ph+ ALL patients remains unclear, as few data in this particular population are available. Moreover, it is not clear today if CAR T-cells will be a curative option for these patients and the role of subsequent allo-HCT has to be defined [164].

## 6. Discussion

Over the last 20 years, frontline TKIs administration in combination with chemotherapy dramatically improved CR rates, duration and deepness of response and long-term survival of adult patients with Ph+ ALL. In the 2G/3G-TKIs era, Philadelphia chromosome do not confer dismal prognosis anymore [165]. However, side effects in this elderly population limit the feasibility of intensive treatment. To reduce toxicity, several strategies to de intensify chemotherapy backbones have been developed: low dose chemotherapy, steroids plus TKI induction and chemotherapy-free regimen. All these strategies led to high response rates and improvement of survival even in elderly population. However, relapses remain the principal reason of treatment failure. Therefore, despite exciting results of these new strategies, it is not clear how far we can reduce our reliance on chemotherapy. Even if survival benefit of allo-HSCT seems less clear in the context of 2G/3G-TKIs, allo-HSCT followed by maintenance remains the standard of care for young and fit patients in CR1. 

Several questions remain unanswered, the first being the choice of TKI. One pediatric randomized clinical trial evaluated effect of dasatinib versus imatinib and showed superiority of dasatinib over imatinib in term of OS, EFS and cumulative incidence of CNS relapses [166]. In the setting of adult patients, two retrospective studies from Sasaki et al., including one propensity score analysis, suggested superiority of ponatinib + HCVAD over imatinib or dasatinib + HCVAD [167,168]. However, there is to date no prospective available data that compares different TKIs for adult Ph+ ALL patients and no specific guidelines [109]. Prospective randomized trials are currently ongoing (Table 2). The choice of TKI could be guided by the presence of BCR-ABL mutations that can be responsible for treatment failure but there is actually no recommendation for mutation screening at diagnosis. These mutations can be detected at diagnosis in around 40% of patients with Ph+ ALL but at frequency often below the level of detectability by direct sequencing and even NGS, and thus requires more sensitive methods for detection [124,169]. The presence of initial CNS involvement can also guide the choice of TKI. In this population of elderly and comorbid patients, toxicity profile of each TKI, including pleural effusion and hemorrhages with dasatinib, vascular events with nilotinib and ponatinib, has also to be considered. An expert panel of German hematologists and cardiologists proposed specific cardiovascular management for patients who are candidates for ponatinib [170]. 2G/3G TKIs should be preferred over imatinib because of their greater efficacy. To note, the FDA approved imatinib, dasatinib and ponatinib for R/R or intolerant adult patients with Ph+ ALL. There is actually no approval for the use of nilotinib or bosutinib in this setting. The EMA approved imatinib integrated with chemotherapy for newly diagnosed adult Ph+ ALL.

Development of blinatumomab-based chemo free strategies is very exciting but the low number of patients included and the short follow up are insufficient to recommend this strategy in the current practice. The optimal administration scheme of blinatumomab has also to be established. Moreover, CNS relapses are a matter of concern during chemo-free treatments, even in case of well-conducted CNS prophylaxis with repeated lumbar punctures, probably due to a lack of efficacy of these agents in the CNS. Therefore, further prospective trials are needed to elucidate if our reliance on chemotherapy can be abolished. Several prospective trials of blinatumomab in combination with other anti-leukemic agents are ongoing (Table 2).

Allo-HSCT is still standard of care for eligible patients in CR1 [109]. However, its role has become less clear with the advent of 2G/3G-TKIs with lower allo-HSCT rates in these trials. Development of blinatumomab and TKIs-based backbones will led to strongly question the role allo-HSCT during the management of Ph+ ALL even in younger patients. However, further studies are required to discriminate between patients who will benefit from allo-HSCT and others. MRD is a useful tool in Ph- ALL patients to stratify allo-HSCT indication [50,171] but its role is less clear in the setting of Ph+ ALL. Short et al. showed that achievement of CMR at 3 months in Ph+ ALL patients receiving chemotherapy plus a TKI as frontline treatment is associated with superior survival and has stronger prognosis impact than molecular response after induction [165]. However, ideal timing for MRD assessment and cutoff levels for the molecular response (MMR, CMR, log-reduction...) need to be prospectively established in large population of Ph+ ALL patients [172]. Moreover, contrary to the CML setting where attempts for international MRD standardization are ongoing since the middle of the 2000′s, consensus guidelines for the assessment of the e1a2 BCR-ABL transcript in Ph+ ALL have been established only recently by the EuroMRD Consortium [5]. At last, multi-lineage expression of BCR-ABL in Ph+ ALL patients has been recently reported [173,174] with persistence of BCR-ABL in non-lymphoblastic cells after treatment and measurable BCR-ABL signal in more than one third of patients [175,176]. In the GRAAPH-2014 phase 3 clinical trial and a retrospective Japanese study, this BCR-ABL “clonal hematopoiesis” was not associated with poorer outcome or higher relapse risk [175]. Therefore, implementation of new tools for MRD monitoring complementary to BCR-ABL monitoring, as IG/TR MRD, may allow better stratification of patients’ relapse risk to define the optimal post-remission strategy.

The prognostic value of MRD has been pointed out in numerous studies in Ph+ ALL and MRD positivity is associated with shorter EFS and OS in a recent meta-analysis [177]. Treatment of MRD-positive Ph- B-ALL patients with blinatumomab is approved since March 2018 but there is no approval for Ph+ ALL patients. A recent phase 2 trial demonstrated the feasibility of inotuzumab administration for Ph+ ALL patients who did not achieve MRD negativity with conventional therapy or who experienced MRD recurrence [178]. This approach has to be further evaluated.

The role of other prognostic factors should also be clarified as IKZF1 deletions that have detrimental effect on response and survival of Ph+ ALL patients [67]. Their presence has been used to stratify the intensity of the treatment of Ph- B-ALL patients in several clinical trials, as in the GRAALL-B 2014 clinical trial [171] but was not evaluated in the context of Ph+ ALL patients. 

## 7. Conclusions

In conclusion, outcome of Ph+ ALL patients dramatically improved over the past 20 years with the development of imatinib and subsequent 2G/3G-TKIs as well as monoclonal antibodies. Consistent efforts have been made to decrease toxicity to the point of chemo free strategies, allowing managing elderly/comorbid patients with clinical benefit. Treatment strategies for Ph+ ALL patients are rapidly evolving and optimization of available therapies as well as development of new molecules are very promising (Table 2).

## Figures and Tables

**Table 1:** Frontline trials of TKI-based regimens in adult Ph+ ALL patients.

Reference	TKI	Phase	N	Median Age, yr (Range)	CHR Rate, %	Early Death Rate, %	Overall CMR Rate, %	Allo-HSCT Rate, %	OS Rate,%	DFS Rate, %	CIR Rate, %	NRM Rate, %
**Intensive Chemotherapy + TKI**												
Fielding, 2014	I	3	175	42 (16–64)	92	5	NA	60	38 (4 yr)	50 (4 yr)	NA	NA
Chalandon, 2015 ^#^	I	3	133	45 (21–59)	91	6.7	23 *	65	43 (5 yr)	32 (5 yr) ^&^	41.3 (5 yr)	22.6 (5 yr)
Bassan, 2010	I	2	59	47 (19–66)	92	4	NA	63	48 (5 yr)	39 (5 yr)	47 (5 yr)	NA
De Labarthe, 2007 Tanguy, 2013	I	2	45	45 (16–59)	96	4.4	38	51	52 (4 yr)	43 (4 yr)	24 (4 yr)	21 (4 yr)
Daver, 2015	I	2	45	51 (17–84)	93	2	45	30	43 (5 yr)	43 (5 yr)	NA	NA
Fujisawa, 2017	I	2	68	49 (18–64)	95.6	4.4	58 *	63	62 (3 yr)	52 (3 yr) ^&^	12.6 (1 yr)	17.3 (1 yr)
Hatta, 2018	I	2	99	45 (15–64)	97	3	NA	61	50 (5 yr)	43 (5 yr)	15 (5 yr)	33 (5 yr)
Ravandi, 2015	D	2	72	55 (21–80)	96	4	65	17	46 (5 yr)	44 (5 yr)	32 (5 yr)	NA
Ravandi, 2016	D	2	94	44 (20–60)	88	2	NA	43	69 (3 yr)	55 (3 yr)	NA	NA
Kim, 2015	N	2	90	47 (17–71)	91	NA	86	63	72 (2 yr)	72 (2 yr)	24 (2 yr)	25 (2 yr)
Jabbour, 2018 Short, 2019	P	2	86	46 (21–80)	100	0 ^~^	83	20	73 (5 yr)	68 (5 yr) ^&^	NA	10 (5 yr)
**Low intensity chemotherapy + TKI**												
Chalandon, 2015 ^##^	I	3	136	48.6 (18–59)	98.5	0.7	29 *	62	43 (5 yr)	42 (5 yr) ^&^	32.8 (5 yr)	23.7 (5 yr)
Goekbuget, 2021	I	3	127	35 (18–55)	95	3	NA	NA	74 (3 yr)	NA	NA	NA
Rousselot, 2016	D	2	71	69 (59–83)	96	4	24 *	9.8	36 (5 yr)	27 (5 yr) ^&^	54 (5 yr)	29
Chalandon, 2018 Rousselot, 2021 ^§^	N	3	156	47 (18–59.9)	98	1.9	NA	58.3	86 (3 yr)	79.6 (3 yr)	21.3 (3 yr)	NA
Ottman, 2018	N	2	72	65.5 (55–85)	94.4	0.72	58 *	33	47 (4 yr)	42 (4 yr) ^&^	34 (4 yr)	34
**Steroids + TKI induction followed by chemotherapy**												
Chiaretti, 2016	I	2	51	46 (17–59.7)	96	0	NA	42.5	48.8 (5 yr)	45.8 (5 yr)	36 (5 yr)	NA
Foa, 2011	D	2	55	53.6 (24–76.5)	92.5	0	NA	34	69 (20 m)	51 (20 m)	57 (20 m)	NA
Chiaretti, 2021	D	2	60	42 (18.7–59)	97	0	NA	43	56.3 (5 yr)	47.2 (5yr)	29.8 (5yr)	18 (5 yr)
Wieduwilt, 2021	D	2	65	60 (22–87)	95.4	0	NA	20	48 (5 yr)	37 (5 yr)	39 (5 yr)	NA
Sugiura, 2022	D	2	78	44.5 (16–64)	94.5	0	58 *	73.4	80.5 (3 yr)	66 (3 yr) ^&^	23.1 (3 yr)	26.1 (3 yr)
**Chemo-free strategy**												
Based on steroids + TKI												
Vignetti, 2007	I	2	30	69 (61–83)	100	0	NA	39	74 (1 yr)	48 (1 yr)	NA	NA
Papayannidis, 2015	N/I	2	39	66 (28–84)	95	0	NA	NA	44 (3 yr)	28 (2 yr)	NA	NA
Martinelli, 2021	P	2	44	66.5 (26–85)	95.5	4.5	81.8	NA	NA	NA	14.3	NA
Based on blinatumomab												
Foa, 2020 Chiaretti, 2021	D	2	63	54 (24–82)	98	1.6	NA	50	87.8 (2 yr)	79.8 (2 yr)	14 (2 yr)	NA
Advani, 2021	D	2	25	73 (62–87)	92	NA	NA	NA	85 (3 yr)	80 (3 yr)	NA	NA
Short, 2021	P	2	19	62 (34–83)	100	0	87	0	100 (1 yr)	100 (1 yr) ^&^	0 (1 yr)	0 (1 yr)

Abbreviations: TKI: tyrosine kinase inhibitor, N: number of patients, yr: years, m: months CHR: complete hematologic response, CMR: complete molecular response, allo-HSCT: allogeneic hematopoietic stem cell transplantation, OS: overall survival, DFS: disease-free survival, CIR: cumulative incidence of relapse, NRM: non relapse mortality, I: imatinib, D: dasatinib, N: nilotinib, P: ponatinib, NA: not available; * CMR post consolidation; ^#^ Intensive arm; ^##^ Low intensity arm; ^§^ Control arm; ^~^ After protocol amendment; ^&^ EFS.

**Table 2:** Ongoing trials for adult Ph+ ALL patients.

Trial	Condition	Age (years)	Phase	Regimen	N	Primary Outcome Measures
NCT03589326	ND	≥18	3	Ponatinib + RI CT vs. imatinib + RI CT	230	CMR
NCT04530565	ND	18–75	3	TKI + steroids vs. TKI + CT vs. TKI + blinatumomab	330	OS
NCT04722848	ND	≥18	3	Ponatinib + blinatumomab vs. imatinib + CT	236	EFS
NCT03624530	ND	14–65	2/3	Post allo-HSCT maintenance with TKI	82	OS
NCT04688983	ND	≥55	2	Ponatinib + blinatumomab vs ponatinib + CT vs. imatinib + CT	180	MRR
NCT04554459	ND	18–65	2	Ponatinib + RI CT	32	CMR
NCT02776605	ND	18–55	2	Ponatinib + intensive CT	30	ORR, EFS
NCT04329325	ND	≥18	2	Dasatinib + blinatumomab + dexamethasone	17	CMR
NCT04845035	ND	≥18	2	Alternating dasatinib and ponatinib + intensive CT (BFM like)	23	CMR
NCT04747912	ND	≥18	2	Inotuzumab + dasatinib + dexamethasone	25	CR
NCT03541083	ND	18–70	2	Blinatumomab	71	CMR
NCT04375683	ND	18–80	2	Flumatinib + CT	23	CR, CMR
NCT04788472	ND	≥15	1/2	Sequential CD19 and CD22 CAR-T Therapy	50	DLT, AEs
NCT03114865	ND	≥18	1/2	Post allo-HSCT maintenance with blinatumomab	65	OS
NCT05026229	ND	18–65	NA	Dasatinib + RI CT vs. intensive CT (in consolidation)	60	CR, CMR
NCT05024357	ND	18–65	NA	Post allo-HSCT maintenance with dasatinib (6 m vs 1 yr)	80	CMR
NCT03147612	ND, R/R	≥18	2	Ponatinib + RI CT followed by ponatinib + blinatumomab	60	CMR, ORR
NCT01371630	ND, R/R	≥60	1/2	Inotuzumab + RI CT	276	MTD, PFS, ORR, OS
NCT03263572	ND, R/R	≥18	2	Ponatinib + blinatumomab + MTX + cytarabine	60	CMR, ORR
NCT03595917	ND, R/R	≥18	1	Dasatinib + asciminib + prednisone	34	MTD
NCT02143414	ND, R/R	≥65	2	Blinatumomab + POMP	58	OS, DLT
NCT03610438	MRD+ **	≥18	2	Inotuzumab	76	CMR
NCT02458014	MRD+	≥18	2	Blinatumomab	40	RFS
NCT03982992	MRD+ *	≥18	2	Blinatumomab +DLI	12	AEs
NCT03441061	MRD+	≥18	2	Inotuzumab	40	RFS
NCT04475731	MRD+, R/R	≥18	2	Ponatinib (+CT if R/R)	67	CMR
NCT03104491	MRD+, R/R	16–75	1/2	Post allo-HSCT maintenance with inotuzumab	44	MTD, DLT, DFS
NCT04233346	R/R, T315I	≥18	2	Ponatinib	90	MHR
NCT02997761	R/R	≥18	2	Blinatumomab + ibrutinib	20	CR
NCT02311998	R/R	>18	1/2	Bosutinib + inotuzumab	80	MTD, MHR, CR/CRi
NCT03576547	R/R	≥18	1/2	Ponatinib + venetoclax + dexamethasone	38	MTD, ORR
NCT03160079	R/R	≥18	1/2	Blinatumomab + pembrolizumab	24	ORR
NCT03512405	R/R	≥18	1/2	Blinatumomab + pembrolizumab	36	AEs, CR/CRi
NCT03698552	R/R	≥18	1/2	ADCT-602 (CD22-targeting monoclonal antibody)	65	MTD, RP2D, CR/CRi
NCT04260022	R/R	≥18	1b	HQP1351 (Olverembatinib)	62	Cmax/AUC
NCT04872790	R/R	≥18	1	Dasatinib + venetoclax + prednisone + rituximab	20	MTD, RP2D, AEs
NCT03991884	R/R	≥18	1	Inotuzumab + intensive CT (DA-EPOCH)	24	MTD
NCT02081378	R/R	≥18	1	Asciminib alone or + TKI (imatinib, nilotinib, dasatinib)	326	MTD, RP2D
NCT02879695	R/R	≥16	1	Blinatumomab + ipilimumab + nivolumab	30	AEs, MTD
NCT01925131	R/R	≥18	1	Inotuzumab + CT	50	MTD
NCT05016947	R/R	≥18	1	Inotuzumab + venetoclax	26	MTD

Abbreviations: N: number of patients, ND: newly diagnosed, R/R: relapsed/refractory, MRD+: positive molecular residual disease, vs.: versus, RI: reduced-intensity, CT: chemotherapy, BFM: Berlin-Frankfurt-Munster, allo-HSCT: allogeneic hematopoietic stem cell transplantation, POMP: Prednisone, Vincristine, Methotrexate, 6-Mercaptopurine, CR: complete response, CRi: CR with incomplete hematologic recovery, MHR: major hematologic response, CMR: complete molecular response, MRR: molecular response rate, OS: overall survival, EFS: event-free survival, PFS: progression-free survival, RFS: relapse-free survival, RP2D: recommended phase 2 dose, MTD: maximum tolerated dose, DLT: dose limiting toxicity, AEs: adverse events, AUC: area under the curve; * Treatment-resistant mixed chimerism or positive minimal residual disease; ** Before HSCT.

**Table 3:** Outcome of adult Ph+ ALL patients according post remission strategy: allo-HSCT versus no allo-HSCT.

Reference	TKI	Phase	N	Median Age, yr (Range)	Allo-HSCT, %	OS	DFS
Chalandon, 2015	I	3	268	47 (18–59)	63	*p* = 0.02 (5 yr) *	*p* = 0.036 (5 yr) *
Fielding, 2014	I	3	175	42 (16–64)	60	52% vs. 19% (4 yr) *	69% vs. 18% (4 yr) *
Bassan, 2010	I	2	59	47 (19.5–66)	63	42% (5 yr) vs. 29% (3 yr) *	46% (5 yr) vs. 8% (3 yr) *
Tanguy, 2013	I	2	45	45 (16–59)	51	76% vs. 33%; *p* = 0.17 (4 yr) *	71% vs. 33%; *p* = 0.26 (4 yr) *
Daver, 2015	I	2	45	51 (17–84)	30	NA	63% vs. 43%; NS (5 yr)
Chiaretti, 2016	I	2	51	45.9 (15–60)	42	*p* = 0.03 *	*p* = 0.06 *
Lou, 2017	I	2	153	40 (18–68)	39	73% vs. 22%; *p* < 0.0001 (3 yr) *	66% vs. 16%; *p* < 0.0001 * (3 yr) ^#^
Hatta, 2018	I	2	99	45 (15–64)	61	NA	54% vs. 36%; NS (5 yr)
Ravandi, 2015	D	2	72	55 (21–80)	17	33% vs. 49% (5 yr, NS)	NA
Kim, 2015	N	2	90	47.0 (17–71)	63	80% vs. 72%; NS (2 yr)	78% vs. 49%, *p* = 0.045 (2 yr) *
Jabbour 2018	P	2	65	47 (39–61)	20	70% vs. 87%; NS (5 yr)	NA

Abbreviations: TKI: tyrosine kinase inhibitor, n: number, yr: years, allo-HSCT: allogeneic hematopoietic stem cell transplantation, OS: overall survival, DFS: disease-free survival, EFS: event-free survival, IM: imatinib, DA: dasatinib, NI: nilotinib, PO: ponatinib, NA: Not available; * These results support the role of Allo-HSCT in adult Ph+ ALL patients in CR1; ^#^ EFS.
